# A functional variant in miR-155 regulation region contributes to lung cancer risk and survival

**DOI:** 10.18632/oncotarget.5840

**Published:** 2015-10-29

**Authors:** Kaipeng Xie, Hongxia Ma, Cheng Liang, Cheng Wang, Na Qin, Wei Shen, Yayun Gu, Caiwang Yan, Kai Zhang, Ningbin Dai, Meng Zhu, Shuangshuang Wu, Hui Wang, Juncheng Dai, Guangfu Jin, Hongbing Shen, Zhibin Hu

**Affiliations:** ^1^ Department of Epidemiology and Biostatistics, Collaborative Innovation Center of Cancer Medicine, School of Public Health, Nanjing Medical University, Nanjing, China; ^2^ Jiangsu Key Lab of Cancer Biomarkers, Prevention and Treatment, School of Public Health, Nanjing Medical University, Nanjing, China; ^3^ The First Affiliated Hospital of Nanjing Medical University, Nanjing, China

**Keywords:** non-small cell lung cancer, genetic susceptibility, prognosis, miR-155

## Abstract

Emerging evidence suggested that upregulation of miR-155 could serve as a promising marker for the diagnosis and prognosis of non-small cell lung cancer (NSCLC). In the present study, we genotyped rs767649 (A > T) located in miR-155 regulation region in 1341 cases and 1982 controls, and analyzed the associations of rs767649 with NSCLC risk and survival. Consequently, rs767649 exhibited the significant associations with the risk (adjusted OR = 1.12, 95% CI = 1.01–1.24, *P* = 0.031) and prognosis of NSCLC (adjusted HR = 1.17, 95% CI = 1.03–1.32, *P* = 0.014). Meanwhile, rs767649 specifically interacted with radio-chemotherapy (*P_int_* = 0.013), and patients with both the rs767649-TT genotype and radio-chemotherapy had the highest hazard ratio (adjusted HR = 1.65, 95% CI = 1.26–2.16, *P* < 0.001). Furthermore, using functional assays and The Cancer Genome Atlas (TCGA) Lung Adenocarcinoma (LUAD) dataset, we found that rs767649 variant allele could increase the transcriptional activity of miR-155, which in turn facilitated tumor growth and metastasis by inhibiting *HBP1*, *TJP1*, *SMAD5* and *PRKAR1A* expression. Our findings suggested that rs767649 A > T might contribute to the increased risk and poor prognosis of NSCLC, highlighting the importance of rs767649 in the prevention and therapy of NSCLC.

## INTRODUCTION

Lung cancer, predominantly non-small cell lung cancer (NSCLC), is one of the most common malignancies and the leading cause of cancer death globally [1]. Although much progress has been made in diagnosis and treatment of NSCLC, most patients are still diagnosed with inoperable stage, and 5-year survival rate is generally lower than 20% in both the developed and developing world [2]. Thus, there is a strong need to identify reliable biomarkers in the early detection, precise diagnosis and personalized therapy for NSCLC patients.

MicroRNAs (miRNAs) are a class of 21–25 nucleotide (nt) single-stranded non-coding RNAs, which regulate gene expression by base pairing to their target sequences [3]. A number of studies have reported that some key miRNAs are frequently dysregulated in lung cancer, and contribute to lung cancer development and progression by acting as oncogenes or tumor suppressor genes [4, 5]. MiR-155, one of the best characterized miRNAs, is significantly up-regulated in lung cancer tissues, plasma and sputum, and could serve as a promising marker for the diagnosis and poor prognosis of NSCLC [5–11]. However, the intensive roles and mechanism of upregulated miR-155 in NSCLC are poorly understood.

Like protein coding mRNAs, miRNAs have their own regulation elements in the flanking region, and transcription factors may bind to these regions and control the miRNA transcription [12]. Several studies have shown that genetic variants in these functional elements could alter the expression of miRNA and thus contribute to the risk and prognosis of human disease. For instance, our previous study demonstrated that rs928508 located in the pre-miR-30c-1 flanking region significantly decreased the expression of precursor and mature miR-30c and had a significant association with the poor survival of NSCLC [13]. Luo *et al* identified that rs57095329 in regulatory regions of miR-146a decreased the binding to transcription factor Ets-1 and affected the risk of systemic lupus erythematosus [14]. In addition, Bulik-Sullivan *et al* conducted the comprehensive bioinformatics analysis on genome-wide association studies (GWAS) trait/disease-associated genetic variations in miRNA regulome, and reported that the regulatory elements of miRNA also harbored trait/disease-causing genetic variants [15].

In recent years, the Encyclopedia of DNA Elements Project (ENCODE, http://genome.ucsc.edu/ENCODE/) have delineated a comprehensive maps of candidate functional elements in the genome. For example, H3K4me1 and H3K4me3 represent enhancer and promoter positions [16], respectively, while H3K27ac reflects active utilization of the regions [17]. Furthermore, The Cancer Genome Atlas (TCGA, http://cancergenome.nih.gov) consortium released mRNA and miRNA expression profiling in human cancer, which provides a powerful approach to study the relationship between miRNAs and their target genes. Taken together, these bioinformatics tools provided a good opportunity to prioritize functional variants in regulation elements of miRNAs and evaluate their functional roles in cancer development and prognosis.

Thus, we conducted a case-control study including 1341 cases and 1982 controls to evaluate the associations of potentially functional genetic variants in the regulation region of miR-155 with the risk and survival of NSCLC in a Chinese population, and characterized the function relevance of miR-155.

## RESULTS

### Association of rs767649 with NSCLC risk

Using the ENCODE ChIP-seq data (e.g. H3K4me1, H3K4me3 and H3K27ac) in A549 cell line, we identified a putatively functional SNP (rs767649) in miR-155 regulation region, which was located within a peak region of H3K4me1 (Supplementary Figure S1A, see MATERIALS AND METHODS). Then, rs767649 was genotyped in 1341 NSCLC patients and 1982 controls. The demographic characteristics of subjects are described in Supplementary Table S1. The cases and controls were well matched on age and gender (*P*_age_ = 0.972, *P*_gender_ = 0.179). There were more smokers in the case group compared with that in the control group (*P* < 0.001). The genotype distributions of rs767649 between NSCLC cases and cancer-free controls are shown in Table 1. Logistic regression analysis revealed that the minor allele of rs767649 was significantly associated with an increased risk of NSCLC [additive model: adjusted Odds ratio (OR) = 1.12, 95% confidence interval (CI) = 1.01–1.24, *P* = 0.031] after adjusting for age, gender and smoking. Furthermore, stratified analysis was conducted to estimate the association of rs767649 with NSCLC risk by age, gender, smoking and histological subtypes (Supplementary Table S2). However, no significant difference was observed among subgroups (*P* > 0.05 for heterogeneity test).

**Table 1:** Association of miR-155 rs767649 with risk of lung cancer

Genotype	Cases	Controls	Adjusted OR (95% CI)*	P*
rs767649 (A > T)	N (%)	N (%)		
AA	485 (36.2)	773 (39.0)	1.00	
AT	631 (47.0)	933 (47.1)	1.07 (0.92−1.25)	0.373
TT	225 (16.8)	276 (13.9)	1.28 (1.03−1.58)	0.023
Dominant model				
AA	485 (36.2)	773 (39.0)	1.00	
AT/TT	856 (63.8)	1209 (61.0)	1.12 (0.97−1.29)	0.126
Recessive model				
AA/AT	1116 (83.2)	1706 (86.1)	1.00	
TT	225 (16.8)	276 (13.9)	1.23 (1.01−1.49)	0.037
Additive model	—	—	1.12 (1.01−1.24)	0.031

* Logistic regression with adjustment for age, gender and smoking.

### Relationship between rs767649 and NSCLC survival

The characteristics and clinical features of 1001 NSCLC patients are shown in Supplementary Table S3. In the follow-up period, 545 patients died from NSCLC, and 32 died from other causes. For disease-specific survival analysis, the latter was considered as censored data in the analyses. Among 1001 NSCLC patients, there were 695 males (69.4%) and 600 smokers (59.9%). Gender, smoking, surgical status and clinical stage were significantly associated with survival time (Log-rank *P* < 0.05). Notably, patients with chemotherapy or radiotherapy (MST = 25.6 months) had a 27% significantly higher risk of death [hazard ratio (HR) = 1.27, 95% CI = 1.03–1.56, *P* = 0.026], compared with those without chemotherapy or radiotherapy (MST = 30.8 months). However, chemotherapy or radiotherapy had no significant association with the survival time of NSCLC after adjusting for clinical stage (HR = 0.86, 95% CI = 0.69–1.08, *P* = 0.193).

**Figure 1:**
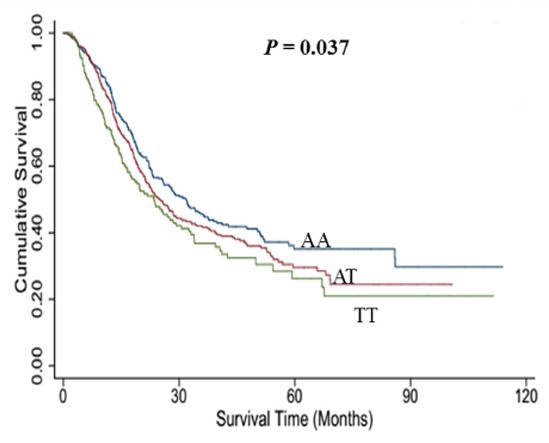
Kaplan-Meier plots of survival by miR-155 rs767649 genotypes in NSCLC

The genotype distributions of rs767649 and its associations with overall survival of NSCLC in different genetic models are presented in Table 2. Log-rank test revealed that rs767649 had a positive association with survival time of NSCLC patients (additive model: *P* = 0.037, Figure 1). Additionally, the results of Cox regression showed that the variant allele of rs767649 was significantly associated with a poor survival after adjusting for age, gender, smoking, surgery status, clinical stage, histological types and chemotherapy or radiotherapy (additive model: adjusted HR = 1.17, 95% CI = 1.03–1.32, *P* = 0.014).

**Table 2:** Associations between genotypes of rs767649 and NSCLC patients' survival

Genotype	Patients *N* = 1001 (%)	Deaths *N* = 545 (%)	MST (Months)	Log-rank *P*	Adjusted HR (95% CI)*	P*
rs767649 (A>T)						
AA	355 (35.5)	181 (33.2)	30.88		1.00	
AT	470 (46.9)	258 (47.3)	24.94	0.145	1.10 (0.91−1.34)	0.331
TT	176 (17.6)	106 (19.5)	23.52	0.011	1.38 (1.08−1.77)	0.009
Dominant model						
AA	355 (35.5)	181 (33.2)	30.88		1.00	
AT/TT	646 (64.5)	364 (66.8)	24.02	0.039	1.17 (0.98−1.41)	0.088
Recessive model						
AA/AT	825 (82.4)	439 (80.5)	27.37		1.00	
TT	176 (17.6)	106 (19.5)	23.52	0.033	1.31 (1.05−1.62)	0.015
Additive model	—	—	—	0.037	1.17 (1.03−1.32)	0.014

Abbreviations: MST, median survival time.

* Adjusted for age, gender, smoking, surgery status, clinical stage, histological types and chemotherapy or radiotherapy.

### Interaction between SNP rs767649 and chemotherapy or radiotherapy

To further characterize the association of rs767649 with NSCLC survival, we performed the stratified analyses by age, gender, smoking, surgery status, clinical stage, histological types and chemotherapy or radiotherapy, and observed the significant heterogeneity in subgroup of chemotherapy or radiotherapy (*P* for heterogeneity test = 0.012, Supplementary Table S4). The results showed that the variants of rs767649 had unfavorable roles in patients with chemotherapy or radiotherapy (adjusted HR = 1.27, 95% CI = 1.11–1.45), but not in patients without these treatment (adjusted HR = 0.84, 95% CI = 0.63–1.13). We explored the interaction between rs767649 genotypes and chemotherapy or radiotherapy status, and observed that rs767649 was specifically interact with chemotherapy or radiotherapy (*P_int_* = 0.013, Table 3). Compared to patients with both AA genotype and chemotherapy or radiotherapy, those with AA or AT genotype but without chemotherapy or radiotherapy had a significantly higher death risk (adjusted HR = 1.74, 95% CI = 1.15–2.63, *P* = 0.009; adjusted HR = 1.60, 95% CI = 1.15–2.24, *P* = 0.006, respectively). In the patients treated with chemotherapy or radiotherapy, we observed the genotype-specific hazard ratios (adjusted HR_AT_ = 1.13, 95% CI = 0.91–1.41, *P* = 0.100; adjusted HR_TT_ = 1.65, 95% CI = 1.26–2.16, *P* < 0.001), suggesting that rs767649 variant allele might identify patients who will not benefit from the treatment of chemotherapy or radiotherapy.

**Table 3:** Interaction between rs767649 genotypes and chemotherapy or radiotherapy on NSCLC survival

Genotype	Chemotherapy or radiotherapy	Patients	Deaths	MST (Months)	Adjusted HR (95% CI)*	P*
AA	Yes	278	145	31.6	1.00	
AA	No	74	33	34.2	1.74 (1.15−2.63)	0.009
AT	Yes	338	192	23.8	1.13 (0.91−1.41)	0.100
AT	No	128	64	28.5	1.60 (1.15−2.24)	0.006
TT	Yes	141	93	19.8	1.65 (1.26−2.16)	<0.001
TT	No	34	13	25.2^§^	1.04 (0.58−1.86)	0.888
*P* for multiplicative interaction	—	—	—	—	—	0.013

Abbreviations: MST, median survival time.

* Adjusted for age, gender, smoking, surgery status, clinical stage and histological types.

§ Mean survival time was present when the MST could not be calculated.

### Allelic difference of rs767649 in miR-155 transcriptional activity

We transiently transfected the luciferase reporter plasmids (rs767649 T or A allele) and pRL-SV40 plasmids into the A549 cell line, and found significantly higher level of luciferase expression in the reporter gene with rs767649 T allele than that with A allele in A549 cell line (Supplementary Figure S1C, *P* = 0.010). The result suggested that rs767649 A > T might upregulate miR-155 expression by increasing the transcriptional activity.

### Effects of miR-155 on the phenotype of lung cancer cells *in vitro* and *in vivo*

**Figure 2:**
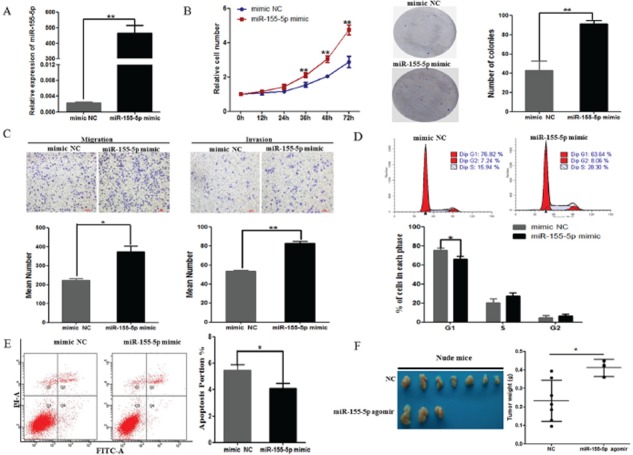
Overexpression of miR-155-5p increased A549 cellular malignant phenotypes **A.** Transfecting miR-155-5p mimics significantly increased the miR-155-5p expression level in A549 cells compared with transfecting a negative control (mimic NC). **B.** Overexpression of miR-155-5p increased cell growth (left) and colony formation (right) of A549 cells. Representative dishes of colony formation in A549 cells are shown. The numbers of colonies were counted and were presented in a histogram. **C.** Representative images (top) and quantification (bottom) of transwell migration (left) and invasion (right) assays in A549 cells after transfection of the negative control or miR-155-5p mimic. **D.** Flow cytometry analysis showed that the cell cycle of A549 cells was decreased at G1 after upregulation of miR-155-5p. The percentage of the cell population at different cell cycle phases is shown in the histogram (bottom). **E.** Effect of miR-155-5p on cell apoptosis assay of A549 cells. The histogram shows the apoptotic cell percentage (right). **F.** Images of tumors (left) from nude mice injected subcutaneously with A549 cells transfected with or without miR-155-5p. Four mice died on day 17, 21, 22 and 36 after inoculation in miR-155-5p agomir group. The mean with SD of the tumor weight is shown (right). *P < 0.05; **P < 0.01. All tests were performed three times independently (A-E).

To further explore the biological significance of miR-155 in lung cancer, we conducted assays of proliferation, migration, invasion, cell cycle and apoptosis in A549 cells. We transiently transfected miR-155-5p mimic or negative control (mimic NC) into A549 cells and found that the level of miR-155-5p was significantly upregulated after mimic treatment (Figure 2A). A549 cells transfected with miR-155-5p mimic exhibited a time-dependent increase in cell viability and more colony numbers compared with the negative control cell (Figure 2B, *P* < 0.05). Meanwhile, transwell assay revealed that the percentage of migrated cells and invasion cells was significantly higher in cells transfected with miR-155-5p mimic (Figure 2C). Furthermore, flow cytometry analyses showed a significant decrease in the number of miR-155-5p mimic cells in G1-phase (Figure 2D), and exogenous miR-155-5p repressed apoptosis (Figure 2E). These results suggested that miR-155 may function as an oncogenic gene in the initiation and progression of lung cancer. Additionally, we evaluated the effect of miR-155-5p on tumor growth in animal models. Consistent with the *in vitro* experiments, our results showed that the weight of tumors from miR-155-5p-regulated xenografts was increased significantly compared with those formed from control xenografts after 6 weeks (Figure 2F, *P* = 0.031).

### Target genes of miR-155-5p signature

**Figure 3:**
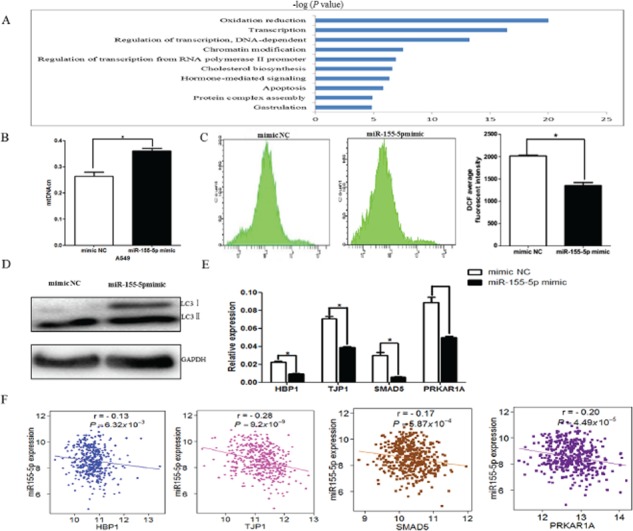
miR-155 regulated oxidative stress-related pathway **A.** The top 10 biological process identified by GO analysis with the target genes. Bars represent −log (*P*-value). **B.** The mtDNA copy number was significantly higher in miR-155-5p mimic-transfected A549 cells as compared with that in the negative control (mimic NC). **C.** Flow cytometry results of the ROS content. x-axis, DCFH-DA fluorescence; y-axis, number of A549 cells. The DCF average fluorescent intensity was also presented in histogram (right). **D.** Western blot analysis of autophagy in A549 cells transfected with miR-155-5p mimic or negative control. LC3 was significantly decreased with increasing miR-155-5p. **E.** The four target genes, including *HBP1*, *TJP1*, *SMAD5* and *PRKAR1A*, which are associated with oxidative stress, were assayed by real-time PCR after overexpression of miR-155-5p in A549 cells. β-actin was used as an internal control. **F.** The negative correlation between the four genes and miR-155-5p expression in TCGA LUAD dataset. All tests (B-E). were performed in triplicate and presented as mean ± SD. *P < 0.05.

Consistent with previous studies, miR-155-5p expression was significantly upregulated in 39 tumor tissues compared with the normal tissues from TCGA LUAD data (*P* = 0.038). To better understand the mechanisms of miR-155-5p in lung cancer, we examined the target genes of miR-155-5p and their involved pathways. First, we applied the *multiMiR* R package [18] to retrieve all the experimentally validated target genes of miR-155-5p and found that 836 genes were potentially regulated by miR-155-5p (data not shown), in which 573 genes were differently expressed in 57 paired lung adenocarcinoma tissues from TCGA (*P* < 0.05, Supplementary Table S5). Then, using the paired miRNA and gene expression data in 420 LUAD tissues, we observed the negative correlation between 129 genes and miR-155-5p expression (Supplementary Table S5). We then conducted GO analysis on these genes and identified that the top associated biologic function was oxidation reduction. Additionally, transcription, regulation of transcription, DNA-dependent, chromatin modification and regulation of transcription from RNA polymerse II promoter as well as apoptosis were also significantly enriched (Figure 3A). Therefore, we first tested if the oxidation reduction was indeed altered in our cell culture models. Interestingly, we found that miR-155-5p increased mitochondrial DNA copy number (mtDNAcn) and reduced reactive oxygen species (ROS) level and LC3II protein expression (autophagy marker), all of which were associated with the oxidative stress (Figures 3B–3D) [19, 20]. Studies have shown that several genes, such as HMG box-transcription protein1 (*HBP1*/*HMGB1*), tight junction protein 1(*TJP1*/*ZO-1*), SMAD family member 5 (*SMAD5*) and the regulatory subunit 1-alpha (RIalpha) of protein kinase A (*PRKAR1A*), were involved in the ROS production or autophagy [21–25]. We further detected the expression levels of four genes in cells transfected with miR-155-5p mimic and negative control. Consistent with the relationships between these genes and miR-155-5p expression in TCGA LUAD data (Figure 3F), our assays confirmed that the expression levels of above genes were significantly reduced in the miR-155-5p-overexpressing cells as compared with that in the control (Figure 3E).

## DISCUSSION

Our findings suggested that rs767649 in the regulation region of miR-155 was associated with the increased risk and poor prognosis of NSCLC, and specifically interacted with chemotherapy or radiotherapy for NSCLC survival. Functional experiments identified that the variant allele might increase the expression of miR-155 and in turn promote cell proliferation, migration and invasion via the oxidative stress-related pathway. Stimulation of tumor formation was also confirmed in the *in vivo* experiment. These findings provide an opportunity to improve the prediction of lung cancer risk and prognosis and optimize treatment strategies for lung cancer patients.

Genetic variants in the functional elements of miRNAs are attracting much attention as they were associated with the risk and survival of various types of cancer [15, 26]. In this study, we did observe that subjects carrying rs767649 variant genotype exhibited a significantly increased risk and poor prognosis of NSCLC, and the T allele increased the transcriptional activity. Interestingly, a previous study has reported that rs767649 was in interferon regulatory factor 2 (*IRF2*) position weight matrix (PWM), and might disrupt the binding of nuclear factor-kappaB (NF-κB) [27]. NF-κB could function as a transcriptional activator that protects lung cancer cells against apoptosis and is a positive mediator of lung cancer cell growth and proliferation as reviewed by Chen *et al* [28]. Therefore, it is biologically plausible that rs767649 variants may bind with NF-κB and active oncogenic miR-155 expression. Meanwhile, we searched several bioinformation databases and found that rs767649 was located in the potential enhancer element in A549 cell line based on ENCODE data and it may also alter regulatory motifs of *IRF*, *MRG1*, *HOXA9* and *PRDM1* using HaploReg v2 based on the 1000 Genomes Project ASN data set (Supplementary Table S6) [29]. We further evaluated the potential function of other SNPs in high linkage disequilibrium (LD) with rs767649 (r^2^ > 0.80) and found that some SNPs were located in regulatory activity regions including promoter, enhancer and DNase hypersensitivity sites, binding proteins and motifs changed. Thus, these SNPs may together contribute to the high expression of miR-155 in lung cancer.

Studies have shown that the miR-155 expression was involved in the sensitivity to chemotherapy or radiotherapy. For example, Zang *et al* identified that the inhibition of miR-155 enhanced the sensitivity of A549 cells to cisplatin treatment [30]. Babar *et al* revealed that increased levels of miR-155 radio-protected A549 cells, while inhibition of miR-155 radio-sensitized these cells [31]. In our study, patients carrying TT genotype of rs767649 had a significantly worse response to chemotherapy or radiotherapy when comparing to those carrying AA genotype and receiving chemotherapy or radiotherapy. Together with the findings that the T allele increased the transcriptional activity of miR-155, we speculated that the T allele may decrease the sensitivity of chemotherapy or radiotherapy via the increased miR-155 expression and result in the unfavorable prognosis.

The oncogenic activity of miR-155 has been recently reported in multiply cancers including lung cancer [32]. One recent study showed that miR-155 acted as an oncogene by targeting 3′UTRs of *SMARCA4* encoding the SWI/SNF catalytic subunit, and had an association with poor prognosis of lung cancer [33]. An animal model study demonstrated that the mice injected with miR-155 tumor cells had an overgrowth of lung tumor [34]. Similarly, we observed that the overexpression of miR-155-5p substantially enhanced the malignant phenotypes of lung cancer cells, including cell growth, colony formation, migration, invasion, and the anti-apoptotic effects. The *in vivo* tumor formation assay in a nude mouse model also demonstrated that miR-155-5p overexpression significantly promoted the tumorigenesis of NSCLC cells compared with the vector control. To explain the possible mechanisms, we identified that the genes potentially inhibited by miR-155 were enriched in the oxidation reduction, which was associated with the production of mtDNA damage, ROS and autophagy. Among these genes, *HBP1*, released by oxidative stress-induced ROS [22], has been recently reported that its decreased expression in A549 cells significantly increased cancer cell migration and invasion *in vitro*, as well as liver metastasis *in vivo* [35]. We observed the decreased ROS level and *HBP1* expression in ectopic expression of miR-155-5p in A549 cells. Therefore, miR-155-5p could promote lung cancer development and metastasis by inhibiting the *HBP1* expression. Additionally, *TJP1* is an epithelial marker associated with tight junctions. Lower expression of *TJP1* was observed in NSCLC tissue and was associated with unfavorable prognosis in NSCLC patients [36]. Furthermore, Yin et al showed that miR-155 inhibited bone morphogenetic protein (BMP)-responsive transcriptional factor *SMAD5* expression in A549 cells, and reversed BMP-induced cell growth inhibition [37]. Taken together, we propose that miR-155-5p could decrease expression levels of these genes and enhance tumor growth and metastasis.

Several limitations need to be addressed in our study. Firstly, we validated these target genes only by real-time PCR, and other detailed functional assays, such as western blot and the luciferase reporter assay, are needed to confirm these findings. Secondly, due to the limited information about disease-free survival, we were unable to evaluate the role of rs767649 in disease-free survival, which might be of great importance in outcome researches. In summary, our study provided evidence that rs767649 in miR-155 contributed to the altered NSCLC risk and survival. Overexpression of miR-155 could promote NSCLC malignant phenotypes, indicating biological significance of miR-155 in lung carcinogenesis.

## MATERIALS AND METHODS

### Study subjects

This study was approved by the institutional review board of Nanjing Medical University. Informed consent was obtained from each subject at recruitment. Since July 2003, a total of 1341 patients with NSCLC have been prospectively recruited from the Cancer Hospital of Jiangsu Province, and the First Affiliated Hospital of Nanjing Medical University, Nanjing, China. All patients were unrelated Han Chinese and histopathologically or cytologically confirmed NSCLC without prior history of other cancers or previous chemotherapy or radiotherapy [38, 39]. The 1982 cancer-free controls were randomly selected from a pool of 30,000 individuals participated in a community-based screening program for non-infectious diseases conducted in during the same time period as the cases were recruited. The control subjects had no history of cancer and were frequency matched to the cases on age and gender. All patients were face-to-face interviewed to collect demographic data and clinical information, including age, gender, smoking, and histological types. Individuals who smoked one cigarette per day for >1 year were defined as ever smokers, otherwise they were considered as never smokers. All patients were followed-up by personal or family contacts from the time of enrollment until death or the last scheduled of follow-up (August 2013). Finally, a total of 1001 cases (74.6%) had sufficient information about survival time to permit statistical analysis with the median survival time (MST) of 26.0 months.

### SNPs selection and genotyping

Based on the UCSC Genome Browser database (http://genome.ucsc.edu/) and the 1000 Genomes database (the Phase I integrated variant set release V3, http://browser.1000genomes.org/index.html), 15 common SNPs (MAF ≥ 0.05 in Chinese Han population) in hsa-mir-155 sequence and its 5′ upstream region (10 kb) were extracted and further annotated by ENCODE activation histone marks peaks (e.g. H3K4me1, H3K4me3 or H3K27ac) in A549 cell line. As a result, rs928883 and rs767649 fell into H3K4me3 and H3K4me1 ChIP-seq regions, respectively (Supplementary Figure S1A). Because rs928883 was in strong linkage disequilibrium with rs767649 (R^2^ = 0.84), only rs767649 was finally selected for genotyping using Sequenom MassARRAY (Sequenom, San Diego, CA, USA). Approximately 5% of the random samples were selected for genotyping repeatedly, yielding a 100% concordance.

### Cell culture

The human lung cancer cells (A549) were purchased from the Shanghai Institute of Biochemistry and Cell Biology, Chinese Academy of Sciences (Shanghai, China) and cultured in completed RPMI 1640 medium (Gibco), supplemented with 10% fetal bovine serum (FBS), 100 U/ml penicillin and 100 μg/ml streptomycin. All of these cells were grown at 37°C with 5% CO_2_. We authenticated the identity of A549, and did not find mycoplasma contamination and cell line cross-contamination.

### Construction of reporter plasmids, transient transfections and luciferase assay

SNP rs767649 is located at 1570 bp upstream from pre-miR-155, and the sequences of pre-miR-155 flanking region (−1770 to −1070) with A or T alleles of rs767649 were synthesized and cloned into *Kpnl* and *Xhol* restrictive sites of the pGL3-promoter vector (Generay Biotech Co., Ltd, Shanghai, China), respectively (Supplementary Figure S1B). The constructed plasmids were sequenced to verify the accuracy. A549 cells were seeded into 24-well culture plates, and were transiently transfected with 500 ng of each constructed vector (with the A allele or T allele) using Lipofectamine 2000 reagent (Invitrogen). Simultaneously, 10 ng pRL-SV40 plasmid per well was co-transfected as the normalizing control for correcting transfection efficiency. After 24 h of transfection, Firefly and Renilla luciferase activities were determined with the Dual-Luciferase Reporter Assay System (Promeg) and expressed as the ratio of Firefly luciferase to Renilla luciferase activities. Three independent transfection experiments were carried out, and each experiment was conducted in triplicate.

### Mimics transient transfection

Human miR-155-5p mimic or negative control (mimic NC) was synthesized by RiBoBio (Guangzhou, China) and transfected into A549 cells using Lipofectamine 2000 reagent (Invitrogen) according to the manufacturer's protocol. After 24 h transfection, cells were isolated for miRNAs and further analyzed.

### RNA isolation and quantitative real-time PCR assay

Total RNA extracted from cells with the Qiagen miRNeasy Mini kit (Qiagen) was reversely transcribed to complementary DNA by using the TaqMan miRNA RT Kit and stem-loop RT primers (Applied Biosystems). MiRNA expression levels were tested using the TaqMan PCR kit as implemented in the ABI 7900 real-time PCR System (Applied Biosystems). To assess the mRNA expression levels of miR-155-5p target genes, SYBR PCR Master Mix reagent kits (TaKaRa) were used according to the manufacturer's instructions. The results of miRNA and mRNA expression were normalized using the threshold cycle (Ct) of U6 and β-actin, respectively. All reactions, including no-template controls, were performed in triplicate. Specific primers for amplification are shown in Supplementary Table S7.

### Proliferation assay

Cell viability of transfected cells was measured by the Cell Counting Kit-8 system (CCK8, Dojindo Laboratory, Japan) according to the manufacturer's instructions. Briefly, 24 h after transfection with miRNAs, cells were collected and reseeded into the 96-well plates at 4000 per well and cultured for 12, 24, 36, 48, 72 h. 10 μl of CCK8 solution was added into each well with 100 μl RPMI 1640 and incubated at 37°C for 2 h. The absorbance was measured at the wave lengths of 450 nm. In colony formation assay, the cells were seeded on 100-mm plate with 600 cells and allowed to grow until visible colonies appeared. Colonies (>50 cells/colony) were fixed in methanol, and then stained with crystal violet (Beyotime). Each assay was performed in triplicate.

### Cell migration and invasion assay

The effects of miR-155-5p on cell migration and invasion were further determined using Costar Transwell plates (6.5 mm diameter insert, 8.0 mm pore size, polycarbonate membrane, Corning Sparks, MD) with or without Matrigel (Falcon BD), respectively. The lower compartment contained 0.6 ml culture medium with 10% fetal bovine serum. 2 × 10^4^ cells in 0.1 ml serum-free medium were added to the upper chamber 1 day for migration and 2 days for invasion prior to the migration experiment. Then, cells were fixed with methanol for 20 min at room temperature, and were stained with 0.5% crystal violet for 30 min. The membranes were then dried, inverted, and mounted on microscope slides for analysis. The cells were counted from five randomly chosen fields per well (× 100) and the mean was determined. All assays were performed in triplicate and repeated three times independently.

### Flow cytometry analyses of cell cycle and apoptosis

Transfected cells were harvested after 48 h transfection. For cell-cycle analysis, cells were washed twice with phosphate-buffered saline (PBS) and then fixed with 70% ethanol overnight at 4°C. Fixed cells were subjected to PI/RNase staining followed by flow cytometric analysis using fluorescence-activated cell sorting (FACS) with a Becton–Dickinson machine (San Jose, CA, USA). Flow cytometric analysis of the apoptotic cells was performed using an Annexin V-FITC/PI staining kit (BD Biosciences) according to the manufacturer's instructions. After washing with cold PBS, the cells were re-suspended in binding buffer, and followed by staining with Annexin V-FITC/PI. The rate of apoptosis was analyzed by flow cytometry (BD Biosciences, San Jose, CA, USA). All of the assays were performed in triplicate and at least three independent experiments.

### *In vivo* tumorigenic assay

All experiments and procedures involving animals were conducted in accordance with the animal care guidelines and protocols approved by Nanjing Medical University animal care unit. Female nude mice (BALB/c), aged 4 weeks, were purchased from the Shanghai Laboratory Animal Center of Chinese Academy of Sciences (Shanghai, China). A549 cells treated with either miRNA analog (agomir) of miR-155-5p (RiboBio) or scrambled miRNA agomir (a negative control) were diluted to a concentration of 2.0 × 10^7^ cells/ml in PBS. Nude mice were injected subcutaneously with 0.1 ml of the suspension into the dorsal flank (7 mice/group). The mice were killed 6 weeks after injection, and the tumor weight was quantified. Four mice died before 6 weeks in miR-155-5p agomir group.

### GO analysis on target genes based on TCGA dataset

All the validated target mRNAs of miR-155-5p were extracted from the *multiMiR* R package [18]. TCGA gene expression data (RNA-seq) for 488 Lung Adenocarcinoma (LUAD) and 57 matched normal samples were downloaded on July 15, 2014 (http://cancergenome.nih.gov/). Then, the differently expressed target genes in paired tumor and normal tissues were chosen. Level 3 miRNA isoform quantification data (reads per million of total reads mapping to a mature microRNA) by miRNA-seq were retrieved from TCGA portal. We filtered out cross-mapped regions and then summed over the reads per million miRNAs mapped (RPM) values for each mature miRNA. After normalization by using the EdgeR package [40], miR-155-5p expression data was extracted from the 420 patients, including 39 paired tumor and normal tissues. All the expression data were log2-transformed. Gene Ontology Enrichment categories of target genes were done by using the Molecule Annotation System (MAS, http://bioinfo.capitalbio.com/mas3/).

### Measurement of intracellular reactive oxygen species (ROS) level

The level of intracellular reactive oxygen species was measured by the Reactive Oxygen Species Assay Kit (Beyotime). The A549 cells of mimic NC and miR-155-5p mimic were collected and exposed to serum-free medium containing 10 μM DCFH-DA. After 20 min of incubation in the darkness, cells were washed with RPMI-1640 for three times, and mean fluorescent intensity (MFI) was measured via flow cytometer with excitation and emission wavelengths of 488 and 525 nm, respectively.

### Analysis of mitochondrial DNA copy number (mtDNAcn)

MtDNAcn was measured using a real-time quantitative polymerase chain reaction and normalized by measurement of the nuclear DNA as previously described [41]. In brief, PCR was performed by amplification of the mitochondrial DNA (NADH dehydrogenase, subunit 1 [*ND1*]) and hemoglobin subunit β (*β-globin*) in nuclear DNA from DNA. The primers of *ND1* gene were as follows: the forward primer 5′-CCCTAAAACCCGCCACATCT-3′ and the reverse primer 5′-CAACTTCATCCACGTTCACC-3′ [42]. The forward primer for *β-globin* was 5′-GAAGAGCCAAGGACAGGTAC-3′ and the reverse was 5′-CAACTTCATCCACGTTCACC-3′ [43]. Each specimen was analyzed in triplicate using 10 ng DNA per 10 μl reaction.

### Western blot analysis

Transfected cells were lysed using the mammalian protein extraction reagent RIPA (Beyotime). After quantification using a BCA protein assay kit (Beyotime), total proteins were separated by SDS-PAGE under denaturing conditions and transferred to PVDF membranes (Millipore). Membranes were blocked in 5% non-fat milk and then incubated with anti-LC3 (1:1000, Sigma, L7543) and anti-GAPDH (1:1000, Beyotime, AG019) respectively. GAPDH was used as an internal control. Protein bands were visualized by using the ECL Plus western blotting detection reagents (Millipore).

### Statistical analysis

The χ^2^ test for categorical variables and Student's *t*-tests for continuous variables were used to examine the difference between cases and controls in the distribution of demographic characteristics, respectively. Genotype frequencies in control group were tested for the Hardy-Weinberg equilibrium by using goodness-of-fit χ2 test. Odds ratios (ORs) and 95% confidence intervals (CIs) were calculated by using logistic regression analyses to evaluate the associations between genotypes and lung cancer risk with adjustment for age, gender and smoking. Log-rank test was used to compare the survival time in different subgroups categorized by patient characteristics, clinical features and genotypes. Univariate and multivariate Cox proportional hazard regression analyses were performed to estimate the crude or adjusted hazard ratio (HR) and their 95% confidence intervals with adjustment of age, gender, smoking, surgery status, clinical stage, histological subtypes and chemotherapy or radiotherapy. The Chi-square-based Q test was applied to test the heterogeneity of associations between subgroups. The paired two-sample Student's *t*-test was used to compare the gene expression or miR-155-5p expression in paired tumor and non-tumor tissues from TCGA dataset. Correlation analysis was performed using Spearman's correlation. Data of functional assays were presented as the mean ± SD and differences were evaluated by Student's *t*-test. All of the statistical analyses were performed with R software (version 3.0.2). All the tests were two-sided and the criterion of statistical significance was set at *P* < 0.05.

## SUPPLEMENTARY FIGURES AND TABLES




