# Supplementary material for: A functional variant in miR-155 regulation region contributes to lung cancer risk and survival
Source: Oncotarget. 2015 Oct 29;6(40):42781–92. doi: 10.18632/oncotarget.5840 (PMC4767470; doi:10.18632/oncotarget.5840)
Supplement: Supplementary file 1 [file oncotarget-06-42781-s001.pdf]

## SUPPLEMENTARY FIGURE AND TABLES

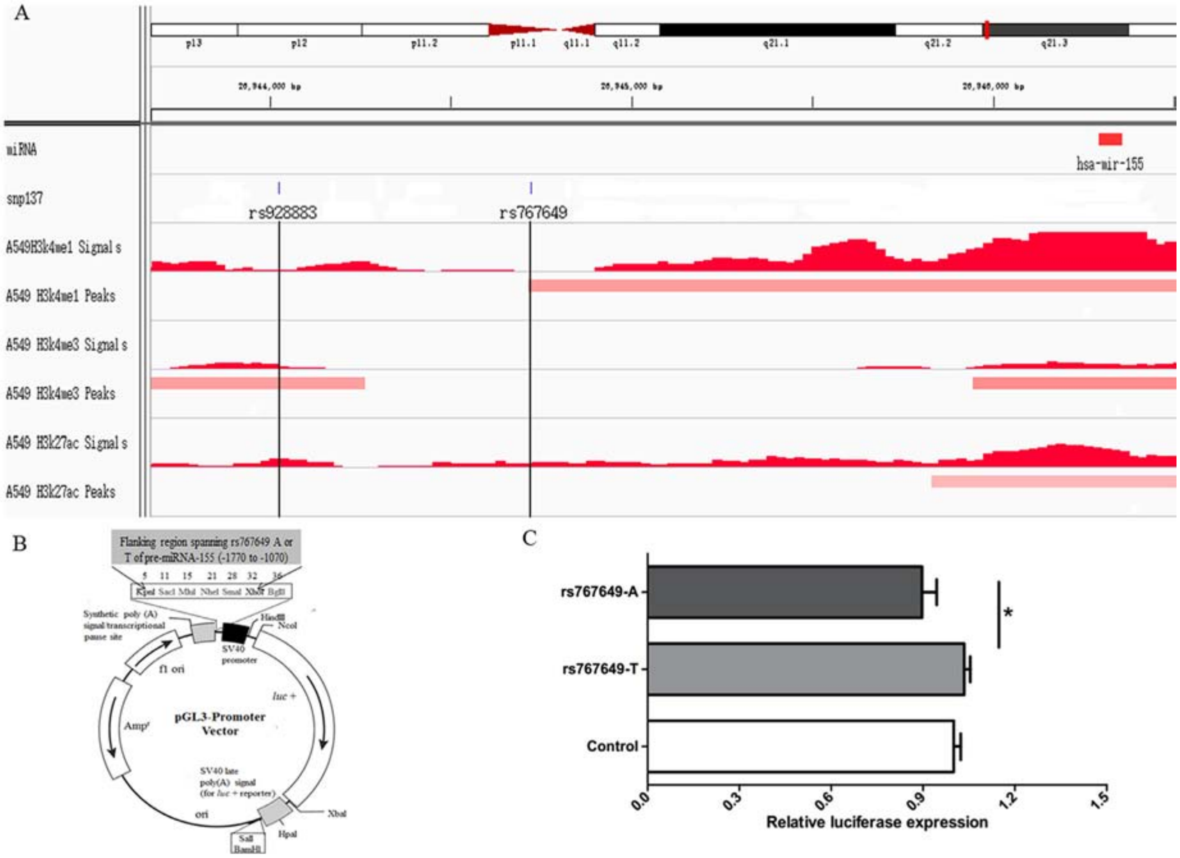


**Supplementary Figure S1: The effect of rs767649 (A > T) genotypes on miR-155 transcriptional level. A.** Functional annotation in proximity to two SNPs (rs928883, rs767649) location in A549 lung cancer cells. The black vertical line highlights the location of the SNPs. The regulation element information include active histone modification marks (H3K4me1, H3K4me3, H3K27ac) from ENCODE project. Peaks of ChIP-seq were reported by the ENCODE project. **B.** Schematic representation of the reporter plasmids construction. **C.** Representative graph of luciferase activity containing rs767649 A or T allele in A549 cells. All constructs were co-transfected with pRL-SV40 to standardize the transfection efficiency. Data presented are the mean ± SD.

**Supplementary Table S1: Characteristics of subjects**

| Variable | Cases (*N* = 1341) | Controls (*N* = 1982) | *P* |
|---|---|---|---|
| | N (%) | N (%) | |
| Age | | | |
| <60 | 589 (43.9) | 873 (44.0) | 0.972 |
| ≥60 | 752 (56.1) | 1109 (56.0) | |
| Gender | | | |
| Male | 949 (70.8) | 1358 (68.5) | 0.179 |
| Female | 392 (29.2) | 624 (31.5) | |
| Smoking | | | |
| Never | 522 (38.9) | 1020 (51.5) | < 0.001 |
| Ever | 819 (61.1) | 962 (48.5) | |
| Histological types | | | |
| Squamous cell carcinoma | 481 (35.9%) | | |
| Adenocarcinoma | 860 (64.1%) | | |

**Supplementary Table S2: Stratified analysis of rs767649 genotypes associated with NSCLC risk**

| Variables | rs767649 (AA/AT/TT) | | Adjusted OR (95% CI)* | *P* for heterogeneity |
|---|---|---|---|---|
| | Cases | Controls | | |
| Age | | | | |
| <60 | 216/266/107 | 327/422/124 | 1.10 (0.95−1.28) | 0.930 |
| ≥60 | 269/365/118 | 446/511/152 | 1.11 (0.97−1.27) | |
| Gender | | | | |
| Male | 339/454/156 | 528/639/191 | 1.12 (0.99−1.26) | 1.000 |
| Female | 146/177/69 | 245/294/85 | 1.12 (0.92−1.35) | |
| Smoking | | | | |
| Never | 196/241/85 | 402/480/138 | 1.05 (0.89−1.23) | 0.496 |
| Ever | 289/390/140 | 371/453/138 | 1.13 (0.99−1.30) | |
| Histological types | | | | |
| Squamous cell carcinoma | 178/225/78 | 773/933/276 | 1.07 (0.91−1.24) | 0.524 |
| Adenocarcinoma | 307/406/147 | 773/933/276 | 1.14 (1.01−1.28) | |

*Adjusted for age, gender and smoking where appropriate in additive model.

**Supplementary Table S3: Patients' characteristics and clinical features in the survival analysis**

| Variables | Patients N = 1001 | Deaths N = 545 | MST (Months) | Log-rank P | HR (95% CI) |
|---|---|---|---|---|---|
| Age | | | | 0.903 | |
| <60 | 428 (42.8) | 229 | 25.8 | | 1 |
| ≥60 | 573 (57.2) | 316 | 27.0 | | 0.99 (0.83−1.17) |
| Gender | | | | 0.034 | |
| Male | 695 (69.4) | 399 | 25.0 | | 1 |
| Female | 306 (30.6) | 146 | 32.9 | | 0.81 (0.67−0.99) |
| Smoking | | | | 0.027 | |
| Never | 401 (40.1) | 197 | 30.0 | | 1 |
| Ever | 600 (59.9) | 348 | 23.9 | | 1.22 (1.02−1.45) |
| Surgical operation | | | | < 0.001 | |
| No | 325 (32.5) | 247 | 14.6 | | 1 |
| Yes | 676 (67.5) | 298 | 44.4 | | 0.33 (0.27−0.39) |
| Clinical stage[a] | | | | < 0.001 | |
| I/II | 417 (41.7) | 165 | 59.3 | | 1 |
| III/IV | 564 (56.3) | 370 | 18.9 | | 2.71 (2.25−3.27) |
| Histological types | | | | 0.060 | |
| Squamous cell carcinoma | 344 (34.4) | 198 | 22.2 | | 1 |
| Adenocarcinoma | 657 (65.6) | 347 | 28.5 | | 0.85 (0.71−1.01) |
| Chemotherapy or radiotherapy[b] | | | | 0.026 | |
| No | 236 (23.6) | 110 | 30.8 | | 1 |
| Yes | 757 (75.6) | 430 | 25.6 | | 1.27 (1.03−1.56)[c] |

[a]Clinical stage information was available in 981 NSCLC patients.
[b]Chemotherapy or radiotherapy information was available in 993 NSCLC patients.
[c]HR = 0.86, 95% CI = 0.69−1.08, $P = 0.193$ after adjusting for clinical stage.

**Supplementary Table S4: Stratified analysis of rs767649 genotypes associated with NSCLC patients' survival**

| Variables | rs767649 (Deaths/Patients) | | | | *P* for heterogeneity |
|---|---|---|---|---|---|
| | AA | AT | TT | Adjusted HR (95% CI)* | |
| Age | | | | | |
| <60 | 83/158 | 94/187 | 52/83 | 1.04 (0.87−1.25) | 0.125 |
| ≥60 | 98/197 | 164/283 | 54/93 | 1.26 (1.07−1.49) | |
| Gender | | | | | |
| Male | 128/244 | 191/331 | 80/120 | 1.20 (1.04−1.39) | 0.619 |
| Female | 53/111 | 67/139 | 26/56 | 1.12 (0.89−1.41) | |
| Smoking | | | | | |
| Never | 76/146 | 88/186 | 33/69 | 1.04 (0.85−1.28) | 0.139 |
| Ever | 105/209 | 170/284 | 73/107 | 1.26 (1.08−1.46) | |
| Surgical operation | | | | | |
| No | 79/99 | 119/164 | 49/62 | 1.15 (0.95−1.40) | 0.791 |
| Yes | 102/256 | 139/306 | 57/114 | 1.19 (1.01−1.40) | |
| Clinical stage | | | | | |
| I/II | 55/150 | 83/197 | 27/70 | 1.06 (0.85−1.32) | 0.329 |
| III/IV | 124/200 | 169/262 | 77/102 | 1.21 (1.04−1.40) | |
| Histological types | | | | | |
| Squamous cell carcinoma | 70/131 | 88/153 | 40/60 | 1.11 (0.90−1.37) | 0.519 |
| Adenocarcinoma | 111/224 | 170/317 | 66/116 | 1.21 (1.03−1.41) | |
| Chemotherapy or radiotherapy | | | | | |
| No | 33/74 | 64/128 | 13/34 | 0.84 (0.63−1.13) | 0.012 |
| Yes | 145/278 | 192/338 | 93/141 | 1.27 (1.11−1.45) | |

*Adjusted for age, gender, smoking, surgery status, clinical stage, histological types and chemotherapy or radiotherapy except for the stratification factor.

**Supplementary Table S5: The correlations between miR-155–5p and differentially expressed target genes based on the TCGA LUAD dataset.**

**Supplementary Table S6: Summary of genomic annotation by HaploReg v2 for SNPs with rs767649 in high LD ($r^2$ > 0.80)**

| CHR | SNP | LD ($r^2$) | Promoter | Enhancer | DNAse | Proteins bound | Motifs changed |
|---|---|---|---|---|---|---|---|
| 21 | rs76667219 | 0.82 | | | | | 4 altered motifs |
| 21 | rs61164219 | 0.83 | | | | | Pax-5,Pax-6 |
| 21 | rs2829789 | 0.99 | | | | | GR |
| 21 | rs17001080 | 0.99 | | | | | Hmbox1,PLZF |
| 21 | rs80143296 | 0.99 | | | | | 7 altered motifs |
| 21 | rs60758621 | 0.99 | | | | | Foxk1 |
| 21 | rs80238897 | 0.99 | | | | | 6 altered motifs |
| 21 | rs926962 | 0.99 | | GM12878 | | | Hoxa5 |
| 21 | rs79610654 | 0.99 | | | | | Pax-4,TCF12 |
| 21 | rs17001116 | 0.99 | | | | | BATF,Pax-4 |
| 21 | rs17001119 | 0.99 | | | | | 7 altered motifs |
| 21 | rs77345347 | 0.99 | | | NT2-D1 | | 7 altered motifs |
| 21 | rs987195 | 1 | GM12878, NHLF | Huvec | | POL2 | |
| 21 | rs928883 | 0.89 | | GM12878, Huvec | | POL2, POL24H8 | 5 altered motifs |
| **21** | **rs767649** | **1** | | **Huvec, GM12878** | **GM06990** | | **Irf, Mrg1::Hoxa9, PRDM1** |
| 21 | rs149321097 | 0.99 | | GM12878 | HAEpiC, HIPEpiC, HNPCEpiC | 4 bound proteins | 11 altered motifs |
| 21 | rs9636772 | 1 | | GM12878 | | POL2 | DMRT1 |
| 21 | rs8132093 | 0.89 | | GM12878, K562 | 15 cell types | 5 bound proteins | 4 altered motifs |
| 21 | rs1893651 | 0.89 | | | Th1, GM06990, GM12865 | 6 bound proteins | 7 altered motifs |
| 21 | rs80170515 | 1 | | | | BCL3, POL2, POL24H8 | 6 altered motifs |
| 21 | rs2298368 | 1 | | | | | Pou2f2 |
| 21 | rs60928262 | 1 | | | | | Foxj2, Pou2f2 |
| 21 | rs67668070 | 0.99 | | | | | 29 altered motifs |

(*Continued*)

| CHR | SNP | LD (r²) | Promoter | Enhancer | DNAse | Proteins bound | Motifs changed |
|---|---|---|---|---|---|---|---|
| 21 | rs74468704 | 1 | | | | POL24H8 | 4 altered motifs |
| 21 | rs60317333 | 1 | | | | | Pax-4 |
| 21 | rs79149405 | 1 | | | | | CTCF,GATA |
| 21 | rs79867059 | 1 | | | Fibrobl | | |
| 21 | rs75153519 | 1 | | | | | HMG-IY |
| 21 | rs80219511 | 1 | | | | | 7 altered motifs |
| 21 | rs146142792 | 1 | | | | | 9 altered motifs |
| 21 | rs79180215 | 1 | | | | | Pou3f2 |
| 21 | rs61100311 | 0.97 | 9 cell types | | 5 cell types | 6 bound proteins | 7 altered motifs |
| 21 | rs60477916 | 0.96 | 9 cell types | | FibroP,pHTE | 9 bound proteins | 7 altered motifs |
| 21 | rs79090681 | 0.97 | 9 cell types | | 4 cell types | TFIIIC110 | Ets |
| 21 | rs75036370 | 0.97 | 9 cell types | | GM12878, Ishikawa, PanIsletD | TFIIIC110 | |
| 21 | rs79531251 | 0.95 | | | | | 7 altered motifs |
| 21 | rs57384518 | 0.95 | | | | | 8 altered motifs |
| 21 | rs77496800 | 0.95 | | | | | Evi-1,Osf2 |
| 21 | rs74978529 | 0.94 | | | | | |
| 21 | rs78684248 | 0.94 | | | | | HNF4 |
| 21 | rs10432771 | 0.95 | | | | | 5 altered motifs |
| 21 | rs10432836 | 0.95 | | | | | Foxa,Foxi1 |
| 21 | rs78945457 | 0.91 | | | 17 cell types | | 6 altered motifs |

CHR, chromosome; the LD information were derived from the 1000 Genomes Project ASN (East Asian) data for the rs767649 (marked in bold) and its surrogates. Promoter, Enhancer, DNAse, Proteins bound and Motifs changed demonstrated evidence of histone modifications, Dnase hypersensitivity sites or transcription factor occupancy as shown by the HaploReg v2 analysis.

**Supplementary Table S7: Primers for PCR amplification**

| Name | Sequence |
|---|---|
| TJP1 F | 5′-CGAGTTGCAATGGTTAACGGA-3′ |
| TJP1 R | 5′-TCAGGATCAGGACGACTTACTGG-3′ |
| SMAD5 F | 5′-TTGGTGGAGAGGTGTATGCGGAAT-3′ |
| SMAD5 R | 5′-ACAGATTGAGCCAGAAGCTGAGCA-3′ |
| HBP1 F | 5′-TGAAGGCTGTGATAATGAGGAAGAT-3′ |
| HBP1 R | 5′-CATAGAAAGGGTGGTCCAGCTTA-3′ |
| PRKAR1A F | 5′-GTTTTCGGTCTCCTTTATCGC-3′ |
| PRKAR1A R | 5′-TGCTCTCGGTGTTCCATAAATC-3′ |
| β-actin F | 5′-GAAATCGTGCGTGACATTAA-3′ |
| β-actin R | 5′-AAGGAAGGCTGGAAGAGTG-3′ |
